# Dataset on the effects of psychological care on depression and suicide ideation in underrepresented children

**DOI:** 10.1038/s41597-024-03130-5

**Published:** 2024-03-19

**Authors:** Xuerong Liu, Wei Li, Jie Gong, Qianyu Zhang, Xiaobing Tian, Ji-Dong Ren, Lei Xia, Yanyan Li, Yu Zhan, Jing-Xuan Zhang, Hu Chuan-Peng, Ji Chen, Zhengzhi Feng, The Psychological Health Guard for Children and Adolescents Project of China (CPHG) Group, Yue-Guang Liu, Xian-Yong An, Xiang Yuan, Yi Zhang, Jian Yang, Wan-Xia Li, Yao Peng, Yu-Jia Liao, Si-Yu Cheng, Lu Liu, Hong-Jiang Yu, Tian-Tian Liu, Li-Jun Liang, Meng-Zhu Cheng, Xi Zhao, Xiang-Yu Deng, Hui-Lei Xu, Xue-Hua Li, Yi-Han Wen, Jun Lei, Xiao He, Hong-Ying Liu, Lei Zhang, Zhen-Mi Liu, Xiandong Meng, Xia Jiang, Yuan-Yuan Li, Jiajun Xu, Zhiyi Chen

**Affiliations:** 1 Experimental Research Center for Medical and Psychological Science (ERC-MPS), School of Psychology, Third Military Medical University, Chongqing, 400038 China; 2 Nanchong Psychosomatic Hospital (The Sixth People’s Hospital of Nanchong), Nanchong, Sichuan 637000 China; 3 Department of Public Management, Chongqing University, Chongqing, 400044 China; 4 Department of Epidemiology and Public Health Statistics, North Sichuan Medical College, Nanchong, Sichuan 637000 China; 5 School of Psychology, Nanjing Normal University, Nanjing, 518872 China; 6 Department of Psychology and Behavioral Sciences, Zhejiang University, Hangzhou, 310013 China; 7 Department of Children and Maternal Health, Western China Hospital, Sichuan University, Chengdu, Sichuan China; 8 Mental Health Center, Western China Hospital, Sichuan University, Chengdu, Sichuan China

**Keywords:** Health policy, Population screening, Rehabilitation

## Abstract

Massive increases in the risks of depressive disorders and the ensuing suicide have become the overarching menace for children/adolescents. Despite global consensus to instigate psychological healthcare policy for these children/adolescents, their effects remain largely unclear neither from a small amount of official data nor from small-scale scientific studies. More importantly, in underprivileged children/adolescents in lower-middle-economic-status countries/areas, the data collection may not be as equally accessible as in developed countries/areas, thus resulting in underrepresented observations. To address these challenges, we released a large-scale and multi-center cohort dataset (n = 249,772) showing the effects of primary psychological healthcare on decreasing depression and suicidal ideation in these children/adolescents who were underrepresented in previous studies or current healthcare systems, including unattended children/adolescents, orphans, children/adolescents in especially difficult circumstances, and “left-behind” and “single-parenting” children/adolescents. We provided all individual data recording the depressive symptoms and suicide ideation that had been collected at baseline (Oct 2022) and half-year follow-up (May 2023) from practicing this psychological healthcare system.

## Background & Summary

**Fig. 1:**
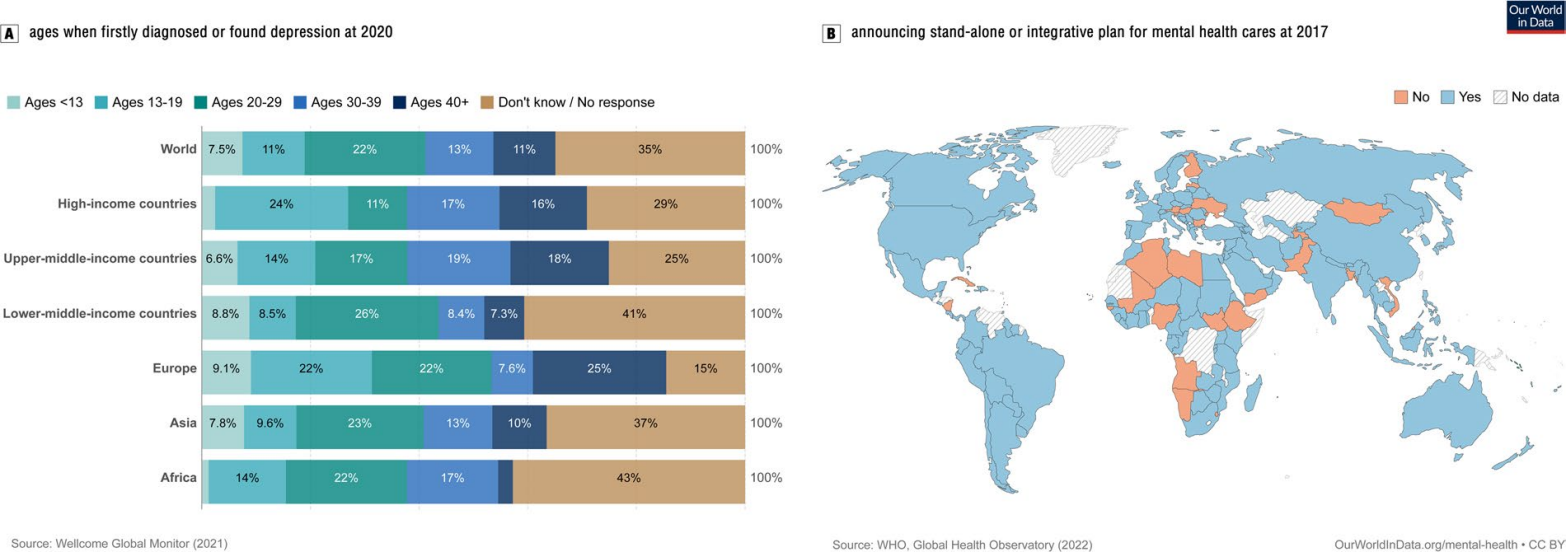
Age pattern of first detecting or diagnosing depressive disorders around the globe (**A**) and country pattern of announcing stand-alone or integrative plans or policies for mental health care in 2017 (**B**). Data and illustrations have been obtained and accessed by Our World in Data repository (https://ourworldindata.org/mental-health).

Given tremendous increases in the incidence of depressive disorders and resultant suicide in children/adolescents, how to stop such unpredictably deleterious outcomes is currently a primary challenge worldwide^1–3^. During the last two decades, the global incidence of depression, particularly in children/adolescents, has increased by more than 20%, while the burdens of such diseases have sharply increased in lower-middle-income countries/areas in recent years (see Fig. 1a)^4–6^. In China, almost one-fifth of adolescents/children have suffered from depressive disorders in the last three years, with nearly 30% of adolescents/children appearing to have major depressive symptoms^7,8^. To make matters worse, suicide relating to depressive disorders has become one of the leading causes of death among children/adolescents in the world, with approximately 11 children/adolescents for suicide death per 0.1 million people^9–12^. To tackle such overarching health threats in children/adolescents, efforts to establish a stand-alone or integrative primary psychological healthcare system have been conducted by the global health-care workforce, especially in globally united organizations and local governments (see Fig. 1b)^13,14^. However, as one of most promising solutions, we know a little neither for whether such systems are practically workable nor for whether the effects of carrying on such health-care systems are stably effective in decreasing risks of depression and suicide for children/adolescents.

The primary psychological healthcare system aims to directly provide universally accessible psychological services or even mental health care for all humans, especially in vulnerable populations (e.g., children and adolescents)^15^. For instance, the United Nations International Children’s Emergency Fund, in conjunction with the World Health Organization and World Bank (WB), has recently instigated the “The Measuring Mental Health Among Adolescents and Young People at the Population Level” (MMAPP) project for offering global psychological care by developing culturally adaptable and clinically validated tools measuring/forewarning their mental health problems (e.g., depression, suicide ideation) (https://data.unicef.org/topic/child-health/mental-health/mmap). In addition, in China, government authorities sponsored a large-scale mental health care reform entitled the “Healthy China 2030 Plan”, which would build upon the primary psychological health care system for screening mental health problems covering nationwide school students per year, particularly in depression and suicide ideation^16,17^. Despite such promising and commendable policies, challenges in the unknown real-world effects and these unexpectedly practical inequities are still imperative to be addressed.

One primary concern worth noting is that such nonprofit medical actions (i.e., primary healthcare) and unpaid incentives for the health-care workforce practically motivate nothing for high-quality services^18,19^. Concerning the current primary healthcare system, to pursue equally accessible medical resources, the primary psychological healthcare systems do not encourage high-quality-but-high-costing care, which lead the effects of such efforts to be unpredictable^20^. More importantly, unexpected inequities in such systems may be brought by multifarious underprivileged conditions, especially in children/adolescents. Despite increases in receiving mental health care in the United States, female, nonwhite, low-middle-income and school-dropped children/adolescents are still found to have fairly less access to such antidepressant services or care^20–22^. Unlike in developed countries/areas, lacking mental health knowledge/educations in low- and middle-income countries (LMICs) may sharpen such inequities for underprivileged children/adolescents who were largely underrepresented from current healthcare system^23^. Although promising and opportune practices in primary psychological healthcare, we know very little about whether such efforts equally benefit these underrepresented cohorts that were exposed from multifarious underprivileged conditions^24,25^.

**Fig. 2:**
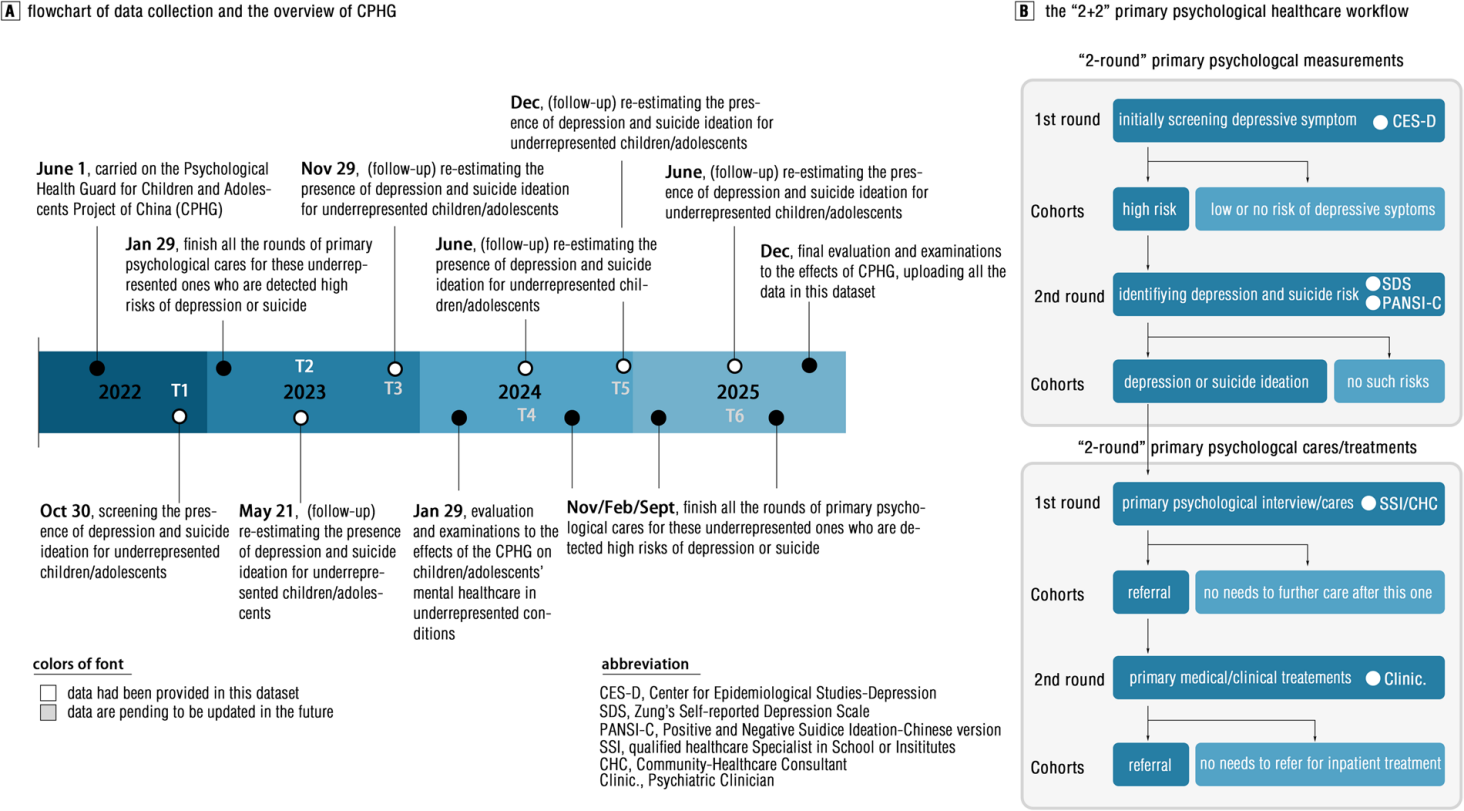
Flowchart of how this dataset was generated and overview of the CPHG. We briefly introduced the main workflow of this “2 + 2” primary psychological healthcare system.

Taken together, the present article shared a dataset showing the effects of primary psychological healthcare on decreasing depression and suicidal ideation for these underrepresented children/adolescents who are commonly suffering from underprivileged conditions in a lower-middle-economic-status area of China (Nanchong, Sichuan) by a prospective, multicenter, preregistered and longitudinal cohort study. Underprivileged conditions requiring legally mandated social care in children/adolescents were defined by the Ministry of Civil Affairs of the People’s Republic of China (MCA, PRC) in the current study, including five cohorts: de facto unattended children/adolescents (dfUCA), orphan, children/adolescents in especially difficult circumstances (CECD), “left-behind” and “single-parent” children/adolescents. They are generally underrepresented from general populations in the psychological healthcare system, as results of poor parenting or family cares. The main data included measurements of depressive symptoms and suicidal ideation after primary psychological healthcare at half-year follow-up (see Fig. 2a) Details for how to implement “2 + 2 model” primary psychological healthcare that the current study deployed can be found in Method section underneath (see Fig. 2b). The overarching goal of releasing this dataset was to help both the scientific community and policymakers (particularly in “Third World” nations/areas) reconsider or even tailor effective and specific primary psychological healthcare systems as early as possible by providing this empirical evidence, favoring universally equal health-care benefits to these underrepresented children/adolescents who are in underprivileged circumstances.

## Methods

These underrepresented children and adolescents aged 6 to 18 years were initially enrolled by retrieving citywide digital unique identifiers (UIDs) from the resident social care system and school registration system, underscoring sampling gender equality as well (n = 313,201, 48.8% girls). To pursue equal access for all underrepresented individuals who are currently in underprivileged conditions, we screened these targeted cohorts outside family, school and social care systems by being aided from local civil-affair authorities. Children/adolescents were finally gathered into the dataset only if the informed consent for agreements on this study had been digitally signed by their parent(s) or legal caregiver(s). All the actions to carry out primary psychological healthcare for these underrepresented cohorts were additionally overseen by local civil-affair authorities. This study has been officially approved by the IRB of Nanchong Psychosomatic Hospital (No. NCPP 2022002).

Underprivileged conditions herein covered almost all the circumstances requiring legally mandated social care in China and were finally checked by local social-care authorities. The dfUCA has been legally defined for child/adolescent whose parent(s) meet the criteria of severe disability, severe illness, serving a prison sentence, mandatory isolation for drug rehabilitation, subject to other measures restricting personal freedom, missing, revoked guardianship qualifications, or being deported (expelled) from the country. The orphans were marked for receiving social care if they lost parents or were unable to locate to specifically biological parents. The CECD referred to an extremely disadvantageous living environment that children/adolescents were experiencing in the family, containing a main member suffering from old age, physical weakness, living alone without care, loss of working capacity, or low economic income that was less than the local minimum criteria that the National Bureau of Statistics of China published per year ($ 8 206 at 2022 in Sichuan). The “left-behind” children/adolescents were identified if they were left in the home without care from either parent. Finally, the “single-parent” ones would be included in the social care system if they were solely parented by a (biological) mother or father. In total, 249,772 children/adolescents with underprivileged conditions that were legally defined above were finally collected into this dataset. They are generally underrepresented from current healthcare system and even existing scientific studies.

### Procedure

The project was conducted by a total of 569 centers for carrying out primary psychological healthcare, with each school or community-based institute (e.g., orphanage, community-care site and children’s hospital) for building a data center. This primary psychological healthcare used a “2 + 2” workflow, with two rounds of psychological screening and two rounds of early psychological care (see Fig. 2b). All the included children/adolescents were initially screened for depressive symptoms by the Center for Epidemiological Studies-Depression Scale (CES-D) (i.e., 1^st^ round to screening, see details underneath). Those who captured risks of depressive symptoms appearing by the last round were tested by both Zung’s self-report depression scale (SDS) and the Chinese version of the Positive and Negative Suicide Ideation Inventory (PANSI-C) (i.e., 2^nd^ round to screening depression and suicide ideation, see details below). To reduce the risks of errors in manual statistics, all these measurements were performed by a purpose-built app that was feasible for multifarious cellphones from mainstreaming manufacturers (e.g., Android, iOS). For further psychological health-care rounds, children/adolescents who were identified with the presence of depression or suicide ideation underwent the first round of psychological care conducted by qualified psychological health-care specialists. A portion of these children/adolescents would be further transferred to a government-sponsored mental health center (hospital) to receive clinical medical treatments if this decision was made by their specialists, which was the second round of early psychological care. As primary health-care services, this “2 + 2” workflow used an open-loop framework to press these children/adolescents with high risks of depression and suicide for reaching full medical cares. Thus, we no long provided clinical care, or followed these children/adolescents if they needed inpatient or additional medical treatments.

### Measurements

As the initial screening, we used the CES-D to detect whether children/adolescents exhibited depressive symptoms. This scale consisted of 4-point-Likert-scoring 20 items and is a widely adopted tool for screening depressive symptoms in nonclinical populations, which requires children/adolescents to report the frequency of appearing these depressive experiences within one week, with 0 points for rating “rare days (<1 day)” and 3 points for rating “almost all the week (5–7 days)”^26,27^. Child/adolescent would be marked with the levels of risk of appearing depressive symptoms if her/his total score of all 20 items exceeded 16 points^28^. Details for all the measurements can be found in the Supplementary Table 1.

Both Zung’s SDS and PANSI-C were utilized for (pre) clinically identifying the presences of depression and suicide ideation in the cohorts that identified depressive symptoms from initial screening (i.e., CES-D scores were over 16 points), respectively. The SDS was widely validated for high reliability and validity in screening children/adolescents’ (pre)clinical depression, which consisted of 20 items scored on a 4-point Likert scale as well, with higher total scores for more severe depressive levels^29,30^. To estimate the risks of coexisting suicide ideation in these depressive children/adolescents, the two-dimensional 14-item PANSI-C was deployed, with one facet for testing the positive suicide ideation (6 items, such as “I feel hopeful for my future life”, “I am very confident for my future”) and with another one for testing the negative suicide ideation (8 items, such as “I am a loser and want to suicide”, “I want to suicide when I cannot address troubles myself”)^31^. This tool was demonstrated for high two-factor structural validity and high reliability in screening adolescents’ suicide and even ensuing suicide behavior^32,33^. It should be noted that the PANSI-C was adopted only for children/adolescents aged more than 12 years old given the local ethical restrictions. Despite such protective policy, this restriction could be exempted if the screening for suicide ideation to children outside this age limitation had been proactively required by their parents, with a legally signed disclaimer.

### Sociodemographic data

The sociodemographic characteristics of each child/adolescent included the most fundamental facets, including gender (i.e., girl/boy), age and living area (i.e., urban/rural). This information was collected by digital records from resident/school registration systems and was confirmed by the children/adolescents themselves.

### Socioeconomic status (SES)

This section measured their family socioeconomic status and even subjective SES by 5 items, such as parents’ educational degrees, occupations and total incomes. Categories of degrees and occupations were coded by benchmarks formulated from the National Bureau of Statistics of China. Full details can be found in the Supplementary Table. 1.

### Family parenting data

We collected data regarding their parenting circumstances in the family, except for those with social parenting (e.g., orphan). This domain included 7 items (if applicable), such as the number of offspring, the experiences of separation from parents and subjective satisfaction for family (see Supplementary Table 1).

### Health-related data

This section collected essential health-related data as legally required by the “Healthy China 2030 Plan” policy. Here, we asked children/adolescents to report their health status (i.e., chronic disease), long-term drug-dependent treatments, sleep and cellphone abuse (see Supplementary Table 1).

### Life events data

This dataset contained 4 items to capture the effects of four major life events on themselves by a 5-point self-rating scale, including “unjustly accused”, “dispute with close friends”, “major traumatic diseases in the family (including yourself)” and “death in the family”. Notably, the life event data were collected from these children/adolescents aged 8–12 years only. Full information can be found in Supplementary Table 1.

## Data Records

A total of 249,772 children/adolescents (48.8% girls) who were underrepresented in such population, were collected in this CPHG dataset, including a de facto unattended cohort (n = 2,444, 49.3% girls), an orphan cohort (n = 762, 44.4% girls), children/adolescents in an especially difficult circumstance cohort (n = 18,419, 45.9%), a “left-behind” cohort (n = 179,877, 48.6% girls) and a “single-parent” cohort (n = 48,270, 50.4% girls). Sociodemographic descriptions can be found in the Supplementary Table 2. By adhering to the “minimum processing” principle, in the Science Data Bank (ScienceDB, 10.57760/sciencedb.12150)^34^, we separately provided a spreadsheet recording these raw data for each cohort mentioned above at each timepoint (T*), one by one. Hence, ten data documents (.xlsx), alongside coding book (.pdf), can be found and downloaded with full access in this repository, with the former five documents for data to the above five cohorts at T1 (Oct 30, 2022) and the latter five documents for these data at T2 (May 21, 2023). For machine-readable architectures, all the file names of these documents would be structured by the framework “cohort ***-timepoint***-Data.xlsx”, such as “Orphan-T1-Data.xlsx”. Details for how to understand the codes and data within each document can be found in the coding book. To enhance the applicability of reusing these data in item-based analyses, the scales/questionnaires originally used in the current study have been provided in this repository.

## Technical Validation

The current study used the multicenter design and thus trained the project-specific staffs by using standardized guidelines. Data collection was implemented or coordinated by trained staffs in each center. To obviate common method bias (CMB) derived from co-measurements in similar resources, we used the one-way Harman’s test for the statistical corrections^35^. The first common factor explained an average of 23.08% (IQR, 21.3–24.5) of the variance across all the variables among these underprivileged cohorts, indicating no statistically significant CMB. Furthermore, all the variables were determined to be feasible by using a low variance filter, which demonstrated that these measurements may be sensitive and workable in screening cohorts. For the psychometric validations, these measurements conducted by scales/questionnaires have also been validated for high internal reliability among these cohorts (i.e., Cronbach’s *α*, mean, IQR, CES-D, 0.949, 0.946–0.950; Zung’s SDS, 0.777, 0.768–0.880; PANSI-C, 0.796, 0.786–0.800). In addition to reliability, high convergent validity was found for these measurements by showing highly positive correlations between homogeneous tests in all cohorts (e.g., CES-D *v.s*. Zung’s SDS, Pearson *r* coefficient, mean, IQR; 0.556, 0.560–0.577, all p < 0.0001), while null correlations of these heterogeneous measurements also revealed high discriminant validity (e.g., CES-D *v.s*. Positive Ideation of PANSI-C, Pearson *r* coefficient, mean, IQR, −0.061, −0.018–0.099, all *p* > 0.5). Finally, post hoc qualitative interviews were carried out with caregivers of a portion of these underrepresented children/adolescents. Ones who detected high risks of depression or suicide ideation from these measurements were all reported for indeed appearing such symptoms or behavioral problems in daily school life by these caregivers, which further indicated the high external validity of these data. Despite high reliability for these measurements, it should bear in mind that no technical detection or corrections (e.g., lie-detection items and time restriction to overly rapid responses) were used in collecting these data. Thus, it may not be guaranteed that the responses for these measurements are all reliable.

## Usage Notes

Privacy and ethical safety are the leading priorities in the present project and the resultant dataset. Despite sharing them with full access, these data are permitted for scientific research and publications only, with full approval that our Data Regulation Office (CPHG-DRO) provided, case-by-case. The application form to this approval can be found in FigShare (10.6084/m9.figshare.24297532.v3). Once this form has been prepared, it could be submitted to the CPHG-DRO (cphg_dro@163.com) for approval. The decision to approve (or not) each application would be made within 10 working days. Unexpected delays may occur if this application is needed for external censorship by the CPHG-DRO. In total, full approval is mandatory once reusing this dataset for scientific purposes.

Analyses of individual children/adolescents are fully forbidden in using this dataset. Due to ethical restrictions in this study, all users should not individually reach out or follow up with any children/adolescents to prevent potential harmful actions. In addition, the usage of this dataset should adhere to local laws, such as the restrictions of using some psychological measurements in children aged below 8 years old in some countries.

Cultural diversity and generalizability should be considered when reusing these data in cross-cultural contexts. A portion of sociodemographic characteristics collected in this study were not applicable or generalizable for all cultures/countries, such as the number of offspring within each pair of parents. To obviate potential biases, this dataset provided raw scales/questionnaires with Chinese that were originally used in the present study. Thus, we strongly recommend that users validate the reliability and validity if they reuse these measurements or materials in the locals.

These cohorts will be totally followed up six times for their depressive symptoms and suicide ideation during 2022–2025 at half-year intervals per investigation (Fig. 2a). We are all committed to keeping this dataset living until all the data that gathered from six follow-ups are released. At the newest update (Jan 2024), the data that collected from first three follow-ups (T1, Oct 2022; T2, May 2023; T3, Nov 2023) have been released in this given repository.

### Supplementary information

Supplementary Table 1

Supplementary Table 2

## Data Availability

The current study did not involve any codes or scripts. Variable codes in this dataset are as follow. The initial of each head of the variable name indicates variable category. Each number alongside from this initial represents specific item in this variable category. Item information can be found in the coding book at the Science Data Bank repository that we provided. D = socioDemographic characteristics; P = family Parenting data; E = family socioEconomic status; L = Living events data; H = Health-related data; C = Center for epidemiologic depression scale data; S = Zung’s Self-reporting depression scale data; SP = Positive Suicide ideation data that measured by Positive and Negative Suicide Ideation scale - Chinese version; SN = Negative Suicide ideation data that measured by Positive and Negative Suicide Ideation scale - Chinese version.

## References

[1] The Lancet (2023). The Lancet’s 200 years: much more to do. Lancet..

[2] Freeman M (2022). The World Mental Health Report: transforming mental health for all. World psychiatry..

[3] Odgers CL, Jensen MR (2020). Annual research review: Adolescent mental health in the digital age: Facts, fears, and future directions. J. Child Psychol Psyc..

[4] Moreno-Agostino D (2021). Global trends in the prevalence and incidence of depression: a systematic review and meta-analysis. J. Affect Disord..

[5] Steffen A, Thom J, Jacobi F, Holstiege J, Bätzing J (2020). Trends in prevalence of depression in Germany between 2009 and 2017 based on nationwide ambulatory claims data. J. Affect Disord..

[6] Ren, X. *et al*. Burden of depression in China, 1990–2017: findings from the global burden of disease study 2017. *J. Affect Disord*. **268**, 95-101 (2020). 10.1016/j.jad.2020.03.011 32158012

[7] Fu, X. L., Zhang, K., Chen, X. F., & Chen, Z. Y. *Report on the Development of National Mental Health in China (2021-2022)* (China Social Sciences Academic Press, 2023).

[8] Fu, X. L., Zhang, K., & Chen, X. F. *Report on the Development of National Mental Health in China (2019-2020)* (China Social Sciences Academic Press, 2021).

[9] Hawton K (2014). Suicide prevention: a complex global challenge. The Lancet Psychiatry.

[10] Naghavi, M. Global, regional, and national burden of suicide mortality 1990 to 2016: systematic analysis for the Global Burden of Disease Study 2016. *bmj* **364** (2019). 10.1136/bmj.l94 PMC6598639 31339847

[11] Fu XL (2023). Suicide rates among people with serious mental illness: a systematic review and meta-analysis. Psychol. Med..

[12] Bould H, Mars B, Moran P, Biddle L, Gunnell D (2019). Rising suicide rates among adolescents in England and Wales. The Lancet.

[13] Singh OP (2021). Comprehensive Mental Health Action Plan 2013-2030: We must rise to the challenge. Indian J. Psychiat..

[14] The L (2022). Ensuring care for people with depression. Lancet (London, England).

[15] Remschmidt H, Belfer M (2005). Mental health care for children and adolescents worldwide: a review. World Psychiatry.

[16] Blakemore S-J (2019). Adolescence and mental health. The Lancet.

[17] Yoon Y, Eisenstadt M, Lereya ST, Deighton J (2023). Gender difference in the change of adolescents’ mental health and subjective wellbeing trajectories. Eur. Child & Adoles Psy..

[18] Li X (2017). The primary health-care system in China. The Lancet.

[19] Li X (2020). Quality of primary health care in China: challenges and recommendations. The Lancet.

[20] Mojtabai R, Olfson M (2020). National trends in mental health care for US adolescents. JAMA Psychiatry.

[21] Whitney DG, Peterson MD (2019). US national and state-level prevalence of mental health disorders and disparities of mental health care use in children. JAMA Pediatrics.

[22] Duong MT (2021). Rates of mental health service utilization by children and adolescents in schools and other common service settings: A systematic review and meta-analysis. Adm. Policy Ment Health..

[23] Renwick, L. *et al*. Mental health literacy in children and adolescents in low-and middle-income countries: a mixed studies systematic review and narrative synthesis. *Eur. Child & Adoles Psy*. 1-25 (2022). 10.1007/s00787-022-01997-6 PMC11032284 35570227

[24] Morris AC (2021). Sociodemographic factors associated with routine outcome monitoring: a historical cohort study of 28,382 young people accessing child and adolescent mental health services. Eur. Child & Adoles Psy..

[25] Madsen KB, Hohwü L, Zhu JL, Olsen J, Obel C (2020). Social selection in cohort studies and later representation of childhood psychiatric diagnoses: The Danish National Birth Cohort. Scand. J Public Health..

[26] William LHC, Chung OKJ, Ho KY (2010). Center for epidemiologic studies depression scale for children: psychometric testing of the Chinese version. J. Adv Nurs..

[27] Betancourt T (2012). Validating the center for epidemiological studies depression scale for children in Rwanda. J. Am Acad Child Adolesc Psychiatry.

[28] Sawyer Radloff L, Teri L (1986). 6/Use of the center for epidemiological studies-depression scale with older adults. Clin. Gerontol..

[29] Zung WW, Richards CB, Short MJ (1965). Self-rating depression scale in an outpatient clinic: further validation of the SDS. Arch. Gen Psychiatry.

[30] Liu X, Ma D, Kurita H, Tang M (1999). Self-reported depressive symptoms among Chinese adolescents. Soc. Psychiatry Psychiatr Epidemiol..

[31] Chen W, Yang T, Gao R, Zhang G (2021). The factor structure and psychometric properties of the Chinese version of the Positive and Negative Suicide Ideation Inventory (PANSI-C) in a non-clinical sample of Chinese adolescents. Ann. Gen Psychiatry..

[32] Muehlenkamp JJ, Gutierrez PM, Osman A, Barrios FX (2005). Validation of the Positive and Negative Suicide Ideation (PANSI) Inventory in a diverse sample of young adults. J Clin Psychol..

[33] Chang HJ, Lin CC, Chou KR, Ma WF, Yang CY (2009). Chinese version of the positive and negative suicide ideation: instrument development. J. Adv Nurs..

[34] Liu X.-R., Feng Z.-Z., Chen Z-Y (2024). Science Data Bank.

[35] Lindell MK, Whitney DJ (2001). Accounting for common method variance in cross-sectional research designs. J. Appl Psychol..

